# Nutritional Supplements for Skin Health—A Review of What Should Be Chosen and Why

**DOI:** 10.3390/medicina60010068

**Published:** 2023-12-29

**Authors:** Jacek Januszewski, Alicja Forma, Julita Zembala, Michał Flieger, Magdalena Tyczyńska, James Curtis Dring, Iga Dudek, Kamila Świątek, Jacek Baj

**Affiliations:** 1 Chair and Department of Anatomy, Medical University of Lublin, 20-090 Lublin, Poland; jacek.januszewski000@gmail.com (J.J.); m.tyczynska@onet.pl (M.T.); jcdring@gmail.com (J.C.D.); kk.swiatek21@gmail.com (K.Ś.);; 2 Department of Forensic Medicine, Medical University of Lublin, ul. Jaczewskiego 8b, 20-090 Lublin, Poland; michalflieeeger@gmail.com (M.F.); iga.dudek6@gmail.com (I.D.); 3 University Clinical Center, Medical University of Warsaw, Lindleya 4, 02-004 Warsaw, Poland; zembalajulita@gmail.com

**Keywords:** supplements for skin, vitamin A, vitamin C, vitamin E, vitamin D, cosmeceutical, nutraceutical, curcumin, chlorella, Omega-3

## Abstract

Supplementation of micronutrients is considered to be crucial in the reinforcement of the skin’s barrier. In this paper, 14 nutritional compounds commonly used in food or pharmaceutic industries were analyzed in terms of influencing skin conditions. The major objective of this paper was to provide a narrative review of the available literature regarding several chosen compounds that are currently widely recommended as supplements that aim to maintain proper and healthy skin conditions. We conducted a review of the literature from PubMed, Scopus, and Web of Science until September 2023 without any other restrictions regarding the year of the publication. Ultimately, we reviewed 238 articles, including them in this review. Each of the reviewed compounds, including vitamin A, vitamin C, vitamin D, vitamin E, curcumin, chlorella, Omega-3, biotin,*Ppolypodium leucotomos*, *Simmondsia chinesis*, gamma oryzanol, olive leaf extract, spirulina, and astaxanthin, was observed to present some possible effects with promising benefits for a skin condition, i.e., photoprotective radiation. Adding them to the diet or daily routine might have a positive influence on some skin inflammatory diseases such as atopic dermatitis or psoriasis. Further, UV radiation protection facilitated by some supplements and their impact on human cells might be helpful during chemotherapy or in preventing melanoma development. Further research is needed because of the lack of clear consensus regarding the doses of the described compounds that could provide desirable effects on the skin.

## 1. Introduction

Skin is the first line of defense that protects our bodies against the influence of negative external factors, such as ultraviolet (UV) irradiation, chemicals, and bacterial infection. Proper nutrition is one of many factors that must be considered when maintaining overall skin health. The inadequate nutritional condition has a detrimental effect on both the structural integrity and biological function of the skin. Such a negative impact may result in the formation of an abnormal skin barrier [1]. Consequently, the significance of micronutrients (such as vitamins and minerals) for skin well-being has been verified in cell culture, animal, and clinical studies [2,3,4]. These nutrients are used not only as active compounds in therapeutic agents for alleviating the symptoms of various skin diseases but also as ingredients in cosmetic products [2]. While their importance has been widely characterized, substances utilizing such substances have been limited to antioxidants and stimulating wound healing. It is crucial to conduct further studies in order to reach a better understanding of previously undefined roles of micronutrients alongside developing potential medicaments that alleviate the symptoms of skin diseases (such as atopic dermatitis, psoriasis, or epidermolysis bullosa) and improve barrier functioning [5,6]. This narrative review discusses the beneficial aspects of various supplements that are currently being investigated and, so far, known as beneficial not only for health in general but also for the skin in particular. In this review, we aimed to discuss the knowledge that is so far segregated and needs further research to provide strict conclusions and potential recommendations.

## 2. Aim of the Review and Search Strategy

**Figure 1:**
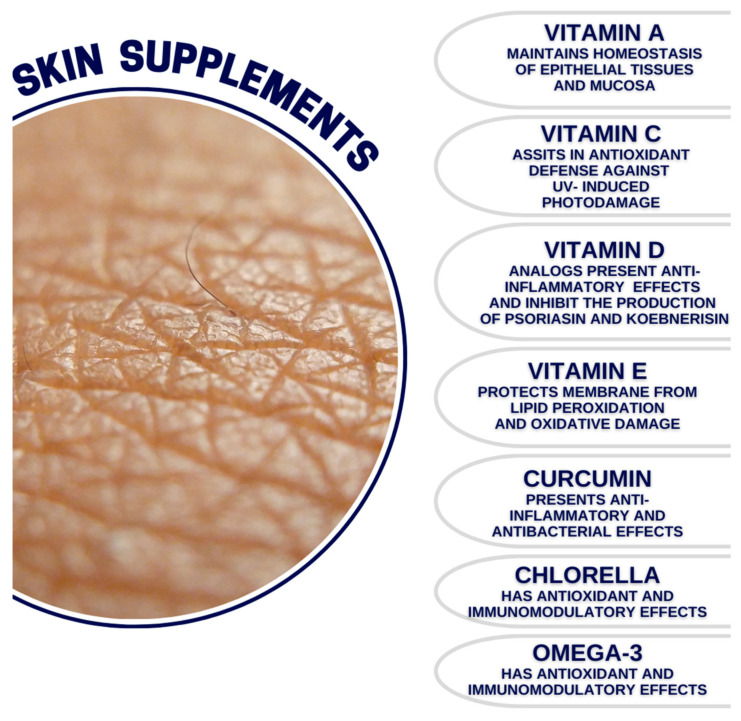
Major properties of the described compounds.

**Figure 2:**
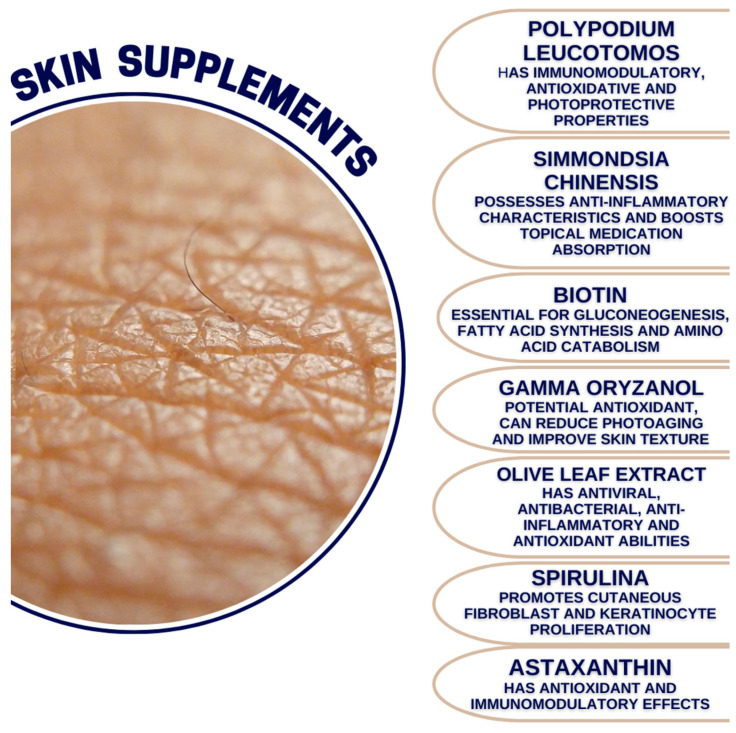
Major properties of the supplements described in the review.

The major objective of this paper was to provide a narrative review of the available literature regarding several chosen compounds that are currently widely sold as supplements that aim to maintain proper and healthy skin conditions. We aimed to discuss research findings concerning each of the described compounds and their usefulness and credibility in terms of the maintenance of the health of the skin. We conducted a review of the literature from three databases, namely PubMed, Scopus, and Web of Science, in September 2023. The first identification of the articles included the search for potential supplements that could be further evaluated in this paper, which was as follows: (skin) AND (supplement OR supplementation). After reviewing the articles devoted to the role of supplementation in various dermatological conditions, we chose the following ones to be included in this narrative review: vitamin A, vitamin C, vitamin D, vitamin E, curcumin, chlorella, Omega-3, *Polypodium leucotomos*, *Simmondsia chinesis*, biotin, gamma oryzanol, olive leaf extract, spirulina, and astaxanthin. The second identification of the articles was conducted using the following search string: (skin) AND (vitamin A OR vitamin C OR vitamin D OR vitamin E OR curcumin OR chlorella OR Omega-3 OR *Polypodium leucotomos* OR *Simmondsia chinesis* OR biotin OR gamma oryzanol OR olive leaf extract OR spirulina OR astaxanthin. Ultimately, we reviewed 238 articles, including them in this review. The literature search was based primarily on human and animal studies. There were no restrictions regarding the year of the publication; all of the review articles were in English. Ultimately, we chose 14 compounds that are currently widely sold in the form of supplements that aim to maintain the health of the skin (Figure 1 and Figure 2).

## 3. Vitamin A

Vitamin A is a lipid-soluble vitamin whose deficiency is linked to impaired immune response and increased susceptibility to skin infections and inflammatory skin diseases. Vitamin A modulates the immune response and maintains homeostasis of epithelial tissues and mucosa through its metabolite, retinoic acid [7]. The said acid controls gene expression through its receptors, which are transcription factors expressed by the skin that mediate the expression of target genes [8]. Deficiencies in vitamin A have been linked to an increased susceptibility to skin infection and inflammation [9]. Seeing as skin epithelium is in direct contact with the external environment, it is exposed to a broad spectrum of pathogens such as bacteria, fungi, and viruses [10]. Keratinocytes, the pro-inflammatory cells of the epidermal layer of the skin, produce antimicrobial proteins (AMPs). AMPs’ function is to kill or inactivate micro-organisms. Epithelial AMPs play a vital role in skin pathogenic invasion prevention [11]. Earlier studies reported that vitamin A deficiency results in immunodeficiency. This state may lead to the predisposition to the increased incidence of *Staphylococcus aureus* (*S. aureus*) skin infection. Evidence indicates that *S. aureus* colonization and vitamin A deficiency are significant in the pathogenesis of atopic dermatitis disease [12]. Patients with atopic dermatitis and defective innate immune response present increased colonization of S. aureus, which correlates with the low diversity of their skin microbiome [13]. Recent works proved that the Resistin family of proteins has anti-bacterial properties. Human Resistin can kill bacteria in the skin at low micromolar concentrations. Mice that do not express Resistin-like molecule α (RELMα) are more prone to skin infection and have an altered skin microbiome. Vitamin A supplementation is required for RELMα expression in mice and rodents treated with the vitamin analog isotretinoin, which had increased expression of RELMα and improved resistance to skin infection [14]. Further investigation of the regulatory role of vitamin A and its by-products on skin immunity, its microbiome, and the development of inflammatory skin diseases should be conducted.

The inclusion of vitamin A, e.g., in skin care products, facilitates the elasticity of the skin and also stimulates the natural process of regeneration of collagen fibers and cellular repair. Not only in creams or serums, but vitamin A is also sold in the form of supplements that have antioxidant effects, prevent viruses and bacteria, and provide a proper condition of skin, hair, and nails, as cosmetic/supplement manufacturers assume. Vitamin A is primarily dedicated to the mature skin with the very first signs of wrinkle appearance. However, it is currently widely used by the youth in order to slow down the process of aging as well as in the case of oily and acne-prone skin since vitamin A is commonly known for its antibacterial and exfoliating properties. Clinical uses of vitamin A and its derivatives, such as isotretinoin, have already been widely used with great success in the treatment of acne vulgaris in many teenagers, and dermatologists would argue that it is the “Gold Standard” starting point for severe acne treatment. Treatment is best recommended at low doses of 20 mg/day for 3–6 months, depending on the severity and the contraindications listed in the guidance [15].

Beta-carotene is an organic pigment abundantly found in plants and fungi. Because of the enzyme *β,β-carotene 15,15’-monooxygenase*, one beta-carotene molecule can be cleaved into two molecules of vitamin A [16]. The average daily intake of beta-carotene is estimated at 2–7 mg [17]. Therefore, beta-carotene is an ingredient that presents high anti-acne and anti-aging properties, facilitating collagen production, cellular regeneration, improvement of skin texture, and acne reduction. In order for the skin to benefit from beta-carotene, which is a precursor of vitamin A, it must be converted in the skin, namely the epidermis, to the retinal and then into retinol. Beta-carotene presents great antioxidant properties, particularly against superoxide radicals and molecular singlet oxygen, being described as much more effective than vitamin E, for example [18]. Further, beta-carotene is involved in the reduction in hyperpigmentation since it stimulates the melanogenesis process [19].

## 4. Vitamin C (L-Ascorbic Acid)

The epidermis, dermis, and hypodermis constitute the three major layers of the skin. For aesthetically healthy skin, the focus leans towards two layers: the epidermal outer layer, which is hypercellular and provides safety function, and the inner dermal layer, which ensures strength as well as elasticity while giving nutritional support to the epidermis. Normal, healthy skin contains high concentrations of vitamin C or L-ascorbic acid, which assist in antioxidant defense against UV-induced photodamage [20]. Additionally, vitamin C benefits human skin physiology with the induction of collagen biosynthesis. The main cutaneous collagens are types I and III, which are produced less with aging. Vitamin C is a common compound used in various skin products, including creams or serums. The effects of its usage in cosmetics include skin firming, prevention of free radicals, stimulation of collagen production, or brightening of the skin. In order to present the aforementioned functions, the cosmetic must include the active, stable formula of the vitamin, primarily vitamin C, in the form of ester. Vitamin C, besides its antioxidant properties, also facilitates sun protection, preventing UVA and UVB. Vitamin C is also a very popular supplement that is primarily taken to facilitate the immune system. However, it also presents additional properties, such as antioxidant properties, that are crucial in the maintenance of healthy skin. Vitamin C is one of the key promoters of collagen formation, but it poorly bypasses the epidermis stratum corneum layer. Scientists observed that squalene vitamin C significantly increases epidermal thickness and preferentially favors collagen III production in human skin, as well as promotes glycosaminoglycan production to a greater extent when compared to free-form L-ascorbic acid [21]. Moreover, L-ascorbic acid inhibits melanogenesis and accelerates wound healing. Vitamin C is a water-soluble molecule, and its transdermal absorption efficiency is low; hence, it is important to recognize efficient transdermal drug delivery methods, such as finding lipophilic derivatives of vitamin C to increase the absorption through the epidermis [22]. The bioavailability of the vitamin in the skin is insufficient with oral intake; therefore, the topical application is used to support surgical healing and improve tissue reconstruction [23]. This way of vitamin C administration reportedly helps with burn wound healing in two aspects. Firstly, it promotes the formation of collagen in skin tissues, and secondly, as an antioxidant, it eliminates free radicals, which further improves the site of the burn wound [20,24]. Vitamin C supplementation modulates inflammatory cytokine secretion, decreases metastasis of melanoma, reduces tumor development, and enhances their encapsulation. Vitamin C is also reported to be effective in case of oily and acne-prone skin. Thus, more in-depth investigation into skin disease management, as well as the potential of vitamin C in dermatology, is needed [25]. Further investigation for topical use in a clinical setting could be arbitrary to aesthetic medicine in the formulation of cosmetic chemical peels to post-treatment procedures to stimulate collagenases and promote wound quicker wound healing.

## 5. Vitamin E

Vitamin E is a lipophilic antioxidant that naturally occurs in eight forms, with the most biologically active being α-tocopherol. Vitamin E, sometimes called the ‘vitamin of youth’, is widely used in cosmetics since it presents antioxidant and anti-inflammatory properties. Regarding cosmetics, primarily, two forms of vitamin E are used, namely alpha-tocopherol and alpha-tocopherol acetate. Vitamin E might also be found in various supplements that aim to facilitate skin conditions from the inside. It was observed that vitamin E deficiency in the diet results in worsening of the skin condition in general and impairments regarding skin appendages, impaired wound healing, or excessive hair loss. Vitamin E’s role is to protect membranes from lipid peroxidation and, therefore, oxidative damage [26]. Studies also highlight the participation of oxidative stress in the pathology of immune-mediated skin diseases, such as atopic dermatitis and psoriasis [27]. The role of vitamin supplementation in these diseases has been investigated. The available clinical data point to a positive role of vitamin E supplementation in both diseases. The study, in which an antioxidant mix of coenzyme Q (10), vitamin E, and selenium was added to the diet of patients with severe forms of psoriasis, was conducted to examine their effect on the disease progression. During the study period, clinical improvement was faster in the supplementation groups than in the control groups. After 30 days, disease severity was significantly lower in the group of patients treated additionally with antioxidant supplementation [28]. Randomized, double-blind clinical research was carried out to assess if vitamin E or D supplementation could improve clinical symptoms in atopic dermatitis patients. Subjects in all groups showed improved symptoms at 60 days compared with baseline, which was measured by the SCORAD index. The most significant improvement was, however, seen in treatment with both vitamins E and D [29]. Another experimental study suggested that vitamin E has antitumorigenic, photoprotective, and skin barrier-stabilizing properties [30]. Clinical evidence is still limited, and, therefore, a standardized approach is needed to create larger trials and expand current knowledge of the vitamin E clinical benefits in atopic dermatitis, psoriasis, or photocarcinogenesis prevention is needed.

## 6. Vitamin D

Vitamin D plays a major role in skeletal and cardiovascular disorders, cancers, central nervous system diseases, reproductive diseases, and infections, as well as in autoimmune and dermatological disorders [31]. Its two main sources are sun exposure and oral consumption, which includes both supplementation and dietary intake. Various factors are associated with vitamin status, particularly skin type, sex, body mass index, physical activity, alcohol intake, and vitamin D receptor polymorphisms. Patients with photosensitive disorders prefer to avoid sun exposure, and because of such practice, they may be at risk for vitamin D deficiency. Maintenance of vitamin D serum concentration within normal levels is necessary for atopic dermatitis, psoriasis, vitiligo, polymorphous light eruption, mycosis fungoides, alopecia areata, systemic lupus erythematosus (SLE), and melanoma patients [32]. Vitamin D might also be supplemented externally in various supplements or medicaments, but it is also found in cosmetics primarily in the form of (either diluted or not) oil solution (alcoholic sucrose or glycol type) but also in the form of capsules for external use. Supplementation of vitamin D (either external or internal) might be highly useful for sensitive, capillary, and mature, aging skin. Polymorphisms of vitamin D receptors are higher in atopic dermatitis patients, which suggests a vital role of vitamin D in the pathogenesis of the disease [33]. The human antimicrobial peptide LL-37 is often overexpressed in skin injury to induce re-epithelialization, and subjects with atopic dermatitis typically have low LL-37 levels. Vitamin D supplementation increased LL-37 expression in atopic dermatitis patients and lowered disease severity in randomized controlled trials [34]. Topical vitamin D analogs present anti-inflammatory and antiproliferative effects, such as IL-2, IL-6, IL-8, IFN-γ, and IL-10 secretion inhibition and T-cell differentiation stimulation. Moreover, they also inhibit the production of psoriasin and koebnerisin [35]. What is crucial is that the specific metabolism of vitamin D depends on CYP11A1, whose activity is associated with such products as lumisterol or 7-dehydrocholesterol that are involved in the regulation of the protective barrier of the skin as well as control skin immune functions [36,37,38]. Except for the abovementioned enzyme of steroidogenesis, CYP11A1, to be biologically active, vitamin D3 must be activated by cytochrome P450. Another aspect regarding human skin physiology is melatonin and its metabolites that are involved in the processes associated with skin pigmentation and hair growth, as well as the development of various skin cancers, including melanoma and nonmelanoma types [39]. It was shown that melatonin might present promising anti-oxidative properties that could act as a defense system against skin aging [40].

## 7. Curcumin

Turmeric, a spice long known for its therapeutic benefits, has piqued the interest of both the medical/scientific community and culinary fans since it is the primary source of polyphenol curcumin [41]. Curcumin is a pleiotropic substance capable of interacting with a wide range of molecular targets implicated in inflammation [42]. Curcumin is well-known for its anti-inflammatory advantages in the treatment of systemic disorders in a holistic manner; it has a wide range of pharmacological actions, including antioxidant and antibacterial characteristics [42,43,44,45,46]. Curcumin might be supplemented both externally and internally. Curcumin used externally in the form of cosmetics alleviates the inflammatory conditions of the skin as well as facilitates the proper functioning of the sebaceous glands. It also eliminates discolorations, accelerates the healing of scars, wounds, and burns, and soothes irritations. Regarding supplements with curcumin, they are sold either as ‘pure’ curcumin supplements or mixtures of various compounds, e.g., with zinc or selen. Supplementation of curcumin also presents benefits for acne-prone skin as it presents anti-inflammatory properties. Healthy skin is something everyone tries to perfect, though certain diet trends and foods can disrupt the skin’s integrity; consuming high fat and refined sugars affects cytokines and autophagy markers of the skin [47,48]. Studies mentioning high-dose tetrahydrocurcumin (THC), which has higher antioxidant activity, have been shown in trials to overcome these limits [41]. Recent research has looked at the anti-inflammatory and antioxidant properties of THC, a main active metabolite of curcumin, in the treatment of tumors and inflammatory illnesses [49,50,51]. Among the skin problems investigated are psoriasis, pruritus, oral lichen planus, facial redness, and numerous types of skin cancer [52]. Oral turmeric supplementation has been demonstrated to be beneficial to skin health. Such conditions facilitated its anti-inflammatory status, as evidenced by the papers that indicated its prospective applications and are highly appreciated for future exploration and potency [52,53,54,55,56,57]. Among these studies, the dose and other concurrent therapies were interlaced due to successful treatment based on patients’ circumstances; therefore, as previously said, more research is strongly suggested. Cheng et al., 2001 conducted a phase 1 clinical trial to investigate the use of curcumin and skin cancer and discovered the harmful effects of oral consumption (>8000 mg/day) while treating in a dose-dependent way; patients’ precancerous lesions improved histologically [58]. A dose-dependent effect was also discovered at nearly all dosage levels [51,58].

The proliferation of epidermal cells, keratinocyte, and lymphocytes are linked to IL-6 activity [59,60,61]. Such IL-6 production was found to have a pathogenic role in various autoimmune and chronic inflammatory disorders; as a result, targeting IL-6 might be a therapeutic strategy for these diseases. Curcuminoid administration resulted in a considerable decrease in circulating IL-6 concentrations; this impact does not appear to be dose-dependent [59]. Curcumin may potentially act as a TNF inhibitor by directly binding to TNF [62]. Curcumin also suppresses COX-2, STAT, cyclinD1, and nuclear factor kappa-light-chain-enhancer of activated B cells (NF-kB) signaling pathways [63], as well as IL-1b and IL-6 production through inhibiting mitogen-activated protein kinase and NF-kB pathways [61,64]. In 2016, a clinical evidence review was published with new information on dermatological indications such as acne, alopecia, atopic dermatitis, psoriasis, oral lichen planus, vitiligo, and facial photoaging [58]. More extensive and substantial clinical trials are required to determine how turmeric can be utilized orally and topically to alleviate the symptoms of various skin diseases. Furthermore, the development of new curcumin delivery techniques with increased bioavailability and solubility would strengthen its efficiency.

## 8. *Polypodium leucotomos*

*Polypodium leucotomos* (PL, commercial name Fernblock^®^, Cantabria Labs Groups (formally IFC Group), Madrid, Spain) is a compound found in ferns from South America of the Polypodiaceae family. Extracts of this fern have long been used to alleviate the symptoms of a number of skin disorders [65,66]. One of the first publications, dated back in 1974, began the journey to improve the clinical outcome of psoriasis. The results of this case provided a good clinical response when consumed orally. Most recently, further studies have examined PL against other dermatologic diseases. Further reports investigated the clinical efficacy and safety when taken orally and used topically [66,67]; however, over the last 40 years, only 19 human and six basic science studies have been reported. The adverse effects of the extract (gastrointestinal symptoms and pruritus) were mild to severe and were observed in a very limited percentage of participants (16/1016 (2%)) [67]. PL is currently sold in various supplements that aim to protect the skin from oxidative stress as well as free radicals, as it has been reported to present antioxidant properties. In addition, it facilitates the healing of inflammatory conditions of the skin and is assumed to prevent burn damage to the skin, preventing both UVA and UVB.

After digging up previous studies, PL gained popularity within the past 10 years, promoting properties ranging from immunomodulatory and antioxidative to photoprotective [66]. Researchers conducted a randomized, double-blind, placebo-controlled trial on healthy adult men and women aged 18 to 65 with Fitzpatrick skin types I to IV [65,66,67]. According to the relevant research, subjects received 480 mg of PL for 60 days, reflecting a higher overall exposure to the substance. There were no reports of adverse events, as in prior clinical investigations [65,68,69,70,71].

Mechanisms of action have found that the combination of UV radiation and PL intake (300 mg/kg) promotes p53 expression in skin cells compared to UV radiation alone, with the degree of induction reaching 2–4-fold greater than the irradiated control. The tumor suppressor gene p53 is widely known, and its activation may be the mechanism behind genomic protection and anti-inflammatory benefits [72,73]. It may also explain the reduction of radiation-induced proliferation of epidermal cells in UV-exposed mice [74,75]. Other studies have found comparable anti-inflammatory effects when assessing serum TNF-α and iNOS expression, which may be downstream of p53 activation, which is known to reduce inflammatory responses [74,76].

The use of natural or phytochemicals has become increasingly popular in recent years. Dietary supplement comprising sulforaphane (SFN), a sulfur-isothiocyanate found in broccoli, coupled with the proprietary extract Fernblock^®^ XP(FB) (*Polypodium leucotomos* (PL), (Cantabria Labs Group (formerly IFC Group) (Madrid, Spain)), produced from the tropical fern *Polypodium leucotomos*, was tested in vitro for antioxidant/antineoplastic effectiveness and anti-aging action [76,77].

*Polypodum leucotomos* has antioxidant properties in vitro, including superoxide, hydroxy, lipid peroxides, and ROS, and it is hypothesized that these properties reduce UV-induced production of Cyclobutane Pyrimidine Dimers secondary to DNA repair enzymes in skin, resulting in fewer UV-induced DNA mutations. In vitro, the SFN/FB combination suppressed melanoma cell motility, metalloproteinases (MMP-1, -2, -3, and -9 generation), inflammasome activation, and IL-1β secretion more effectively than chemical alone [77,78]. It is proposed that SFN/FB-based supplements be used to prevent skin aging and as adjuvants in the alleviation of symptoms of metastatic melanoma.

For 26 weeks, 50 patients with vitiligo vulgaris were randomly allocated to receive 250 mg oral *P. leucotomos* or placebo three times per day, coupled with NB-UVB twice weekly, in a randomized double-blind placebo-controlled RCT. When compared to the placebo group, the results revealed more repigmentation around the head and neck area when compared to other afflicted portions of the body. Patients with skin types II and III had higher levels of repigmentation [79]. The findings of this study have shown to open up a larger population size and should warrant further investigation into additional skin types, particularly type III–VI.

Skin health and elasticity are important factors in maintaining youth and reducing wrinkles and photoaging; according to the data collected and current knowledge, the use of antioxidants (vitamin C, E) and *Polypodium leucotomos* may play a pivotal role and shift skin care regimen toward utilizing proven phytochemicals.

## 9. *Simmondsia chinensis*

*Simmondsia chinensis* (commonly known as jojoba oil in the cosmetic industry) is a plant native to North America’s Sonora desert known as jojoba [80,81]. As a seed lipid energy store, the jojoba plant makes esters of long-chain alcohols and fatty acids (waxes) [81,82]. The oil from the jojoba plant is the primary biological source of wax esters and has a wide range of potential uses, including dermatology [82,83]. Jojoba possesses anti-inflammatory characteristics and can be used to alleviate the symptoms of a range of skin disorders, including skin infections, skin aging, and wound healing. Jojoba has also been found to have a function in cosmetic formulations such as sunscreens and moisturizers, as well as to boost topical medication absorption [82,84]. Functions in the restoration of injured skin fibroblasts are drawn to the wound site to begin the proliferative phase of healing and matrix deposition, whereas keratinocytes participate in the re-epithelialization phase. Jojoba liquid wax (JLW) significantly speeds wound closure by acting on both fibroblasts and keratinocytes, according to our findings. The mechanism was discovered to be Ca^2+^ dependent and to necessitate the involvement of p38, ERK1/2, and the PI3K-Akt-mTOR pathway. JLW also showed the capacity to stimulate collagen I production in fibroblasts [82,83,84,85].

*Simmondsia chinensis* can be found in various cosmetic products (such as emollients in the form of oil, creams, or serums) and because of various beneficial compounds, such as vitamins A, E, or F, along with saturated and unsaturated alcohols and fatty acids, it was reported to be beneficial to maintain the elasticity of the skin also preventing from inflammatory conditions of the skin. Research was carried out and found interesting utilization of essential oils and antimicrobial activity and cytotoxicity. *Simmondsia chinensis* C.K. Schneid (Jojoba oil) was found to be a carrier oil that enhanced antibacterial action while reducing cytotoxicity, with aloe vera reducing essential oil cytotoxicity by 87.5 percent at 24 h and 85 percent at 48 h [86]. Essential oils revealed improved antibacterial action against pathogens such as *Brevibacterium epidermidis*, *B. linens*, and *P. aeruginosa*, with MIC values ranging from 0.09 to 0.50 mg/mL (and FIC 0.14–0.39) [86,87]. Other research involved its use in antifungal and antiviral activity, though the results were not as significant except for treating Herpes-simplex 1 (HSV-1) [80,88]. The results are indicative of higher doses (EC50 of 0.96 ± 0.4 µg/mL) that blocked HSV-1 plaque formation and that JLW wax presence is required to gain protection against HSV-1 infection. In a comparative study, ElLaithy HM and El-Shaboury KM developed and incorporated a vehicle to drive topical fluconazole in treating *Candida albicans* [89]. Fluconazole formulation in several topical treatments, including a series of Cutina lipogels and a gel microemulsion with Jojoba oil as an oleaginous phase [80,85,89,90,91].

Other topical formulations have been presented; however, this study employs niacinamide, and its physicochemical qualities imply that it may have difficulty penetrating the skin’s barrier. A model developed using a nanogel (treatment with the NIA-loaded nanogel/TE jojoba oil) boosted cell survival by up to 80%, showing the formulation’s protective factor [92]. These outcomes rose to 80%, showing the formulation’s protective impact. These findings imply that the protective effect may be attributable to transethosome’s (TE’s) capacity to permeate epidermal cells and allow for localized niacinamide-free scavenging activities. Oral supplementation has been proven to be beneficial in decreasing non-melanoma skin cancers in patients who have already been diagnosed, and the nanogel formulations may be utilized prophylactically in protecting oneself before neoplasia arises throughout adolescence to the mid-20 s [91,93,94].

A clinical trial involved the use of jojoba oil and the known medication, tazarotene, for treating psoriasis topically. A microemulsion system based on jojoba wax and labrasol/plurol isostearique was created. The microemulsion offered a twofold rise in tazarotene skin deposition, which corresponded to a greater decrease in psoriatic patients. Individuals treated with microemulsion experienced no discomfort, but patients treated with commercial gel experienced redness and inflammation [95].

In the field of aesthetic dermatology, there has been an exponential increase in clinical trials and pilot studies attempting to blend holistic and contemporary medicine. Clay masks containing jojoba oil, for example, might be beneficial for lesioned skin as well as for moderate acne [96]. After 6 weeks of therapy with a clay face mask, a 54 percent mean reduction in total lesion count was seen in this study. Because of early evidence that healing clay jojoba oil face masks can be effective for lesioned skin and moderate acne vulgaris, both inflammatory and non-inflammatory skin lesions were dramatically decreased.

Sun protection should be incorporated in all types of skincare and daily routines for people of all ages all over the world, not only to reduce the possibility of acquiring skin malignancies but also to prevent skin photoaging. Many sunscreens are available as physical or chemical blocks, both of which can cause hypersensitivity or block or alter skin integrity. Touitou E and Godin B. discussed the usage of novel non-permeating sunscreens (NPSUN) that are appropriate for cosmetic and medicinal applications [97]. Here, they explained novel photoprotectors were designed to immobilize UV-absorbing moieties in the chemical backbone of Jojoba oil. A reasonable strategy would be to employ non-permeating sunscreens during direct UV exposure, followed by alpha-tocopherol formulations following short- or long-term sun exposure.

The use of jojoba oil as a molecular vehicle for penetrating the skin with the use of medicines could be the deciding factor not only in inefficacious drug delivery but also in providing extra moisture to aid in protection against certain viral and bacterial pathogens.

## 10. Biotin

Biotin (vitamin B7) is a commonly utilized water-soluble B vitamin that is naturally found in foods and is essential for gluconeogenesis, fatty acid synthesis, and amino acid catabolism [98,99,100]. In both animals and humans, biotin deficiency causes neuromuscular dysfunction, baldness, and dermatitis [99,101].

Biotin might be supplemented internally since it is currently found in various supplements, as it was reported that vitamin B7 facilitates the condition of not only the skin but also of hair and nails. Biotin is also found in various cosmetics dedicated to dry, dehydrated, oily, and irritated skin, as it facilitates the proper functioning of the sebaceous glands. Supplementation appears to be the go-to for boosting skin health at the cellular level, yet considerable worry is directed against “overdosing” and low-cost products. There are numerous over-the-counter supplements that might be accessed without a prescription, and individuals may take one tablet/softgel for each listed “improvement.” Perez-Sanchez et al. criticized the safety profile of dermatological supplements. They noted possible interactions, teratogenicity hazards, a lack of independent third-party testing, a lack of warning labeling, and nutritional “overdosing” in this section. Given the lack of regulation of dietary supplements, physicians must inform patients of the possible hazards [102]. Waqas et al. mentioned that physicians were prescribing biotin for hair, skin, and nails and were not fully aware of interferences with laboratory results [103,104,105,106,107]. The majority of physicians accurately said that there have been no randomized studies demonstrating that biotin helps dermatological diseases and that biotin interferes with thyroid and troponin tests [106,107]. Few people were aware of any interference with b-HCG, hepatitis serology, HIV serology, or vitamin D levels, and 19.5 percent were completely uninformed of any interference [103]. Almost half of the physicians did not instruct patients to stop taking biotin before laboratory tests.

Patel et al. discovered 18 instances of biotin usage for hair and nail alterations. Patients taking biotin supplementation had an underlying pathophysiology for poor hair or nail development in all cases. After receiving biotin, all patients demonstrated clinical improvement. There has been a little study proving the usefulness of biotin. Biotin supplementation may be beneficial in situations of acquired and hereditary biotin deficiency, as well as diseases such as brittle nail syndrome or uncombable hair [103]. Biotin supplementation at higher levels (10,000 to 30,000 g/day) is indicated for people with hereditary enzyme deficiencies [108,109,110]. Brittle nail disease and other underlying hair disorders, such as uncombable hair syndrome, need significantly lower biotin supplementation levels ranging from 300 to 3000 g/day [108,111,112].

Biotin evidence, despite it, is still under-studied for its advantages for hair, skin, and nails; clinical usage is nevertheless advantageous to those who perceive a difference and patient preference.

## 11. Gamma Oryzanol

Gamma oryzanol is a ferulic acid ester of rice bran oil sterols [113,114]. In the United States, it is widely promoted as a sports supplement as well as for decreasing blood cholesterol [115,116]. After mentioning the supplement industry, formulations of such compounds with potential antioxidant and skin anti-aging properties are added. Zeinali et al. experimented with γ-Oryzanol (GO), with potential antioxidant and skin anti-aging properties, and prepared nanoethosomes (NEs) containing GO (GO-NEs), and conducted completely cellular studies on murine melanoma cell line (B16F10 cells) for the first time and discovered its apoptotic capability [117]. Currently, it is a compound widely found in various supplements for external use to facilitate skin conditions. In addition, assessments were made to highlight the potential sun protection factor (SPF) of GO-NEs [118]. Furthermore, a long-term (22-week) study was conducted to examine the ability of GO-NEs to prevent UVB-induced skin cancer in vivo in order to offer a novel cosmeceutical formulation. GO has been shown to have strong antioxidant activity [116,117], anti-inflammatory [119], anti-aging [120], and anticancer [121] capabilities, as well as a beneficial influence on enhancing innate immunological response in humans [121]. Despite the benefits indicated above, the application of GO as a new agent for preventing solar UV-related skin cancer is hampered by its large molecular weight (>500 Da) and hydrophobicity, which limit cutaneous distribution through the stratum corneum (SC) [120].

The effectiveness of GO-loaded nanoethosomal formulation on rat skin was demonstrated by histopathological analysis; superoxide dismutase (SOD) and malondialdehyde (MDA) content were investigated, too, as were the dermoscopic and histological effects of GO-loaded nanoethosomes on skin-wrinkle improvement in healthy people and UV-irradiated rats [120]. The findings revealed even further promise for the usability of GO as a means of transportation for effective delivery to reduce skin irritation and skin neoplasia and improve skin-aging protection and wrinkle improvement. The semi-purified rice bran extracts (GO being one of them) formulated into topical creams and gels not only stimulated the proliferation of human fibroblasts and inhibited MMP-2 activity, but they also improved skin attributes such as hydration, pigmentation, thickness, and roughness, and skin elasticity [119,122]. These results enhance the understanding of topical dermatological products that can be used to reduce photoaging and skin texture; however, further studies would benefit an adequate vehicle and form, such as gels and creams.

GO received little attention despite information on specialized carriers for skin penetration and concentration; other chemicals act synergistically to slow the aging process, lower the incidence of melanoma and other skin malignancies, and diminish pigmentation. Who knows what may be produced if dermatologists and scientists work together to deliver accurate and succinct information?

## 12. Olive Leaf Extract

Olive leaf extract (OLE), an over-the-counter supplement containing polyphenols, oleuropein, and hydroxytyrosol [123,124], is a naturally derived substance from olive leaves. Oleuropein has been shown to have a variety of medicinal effects. It was also discovered to have antiviral, antibacterial, anti-inflammatory, and antioxidant activities that may lower upper respiratory illness (URI) rates [124,125,126]. Olive leaf extract is currently sold either in cosmetics (oils, creams, or serums for topical use) or supplements for daily supplementation. This extract is rich in substances such as flavonoids, vitamin C, or oleic acid, which are beneficial for the skin. It presents anti-inflammatory, anti-viral, and antifungal properties. Also, olive leaf extract provides the proper functioning of the blood vessels. It is also known for its antioxidant activities.

Natural phytochemicals have recently been offered as a potential pharmacological option for rejuvenation therapy. However, efficacy is quite restricted; in this case, a small population pilot study examined 36 people with photoaging skin [123]. The findings showed good promise (Asain/Fitzpatrick’s skin type III-V) with decreased wrinkles, roughness, and skin desquamation just 2 months after the application. A small percentage of individuals had moderate and temporary acneiform eruptions after beginning to use the cream. Thus, further investigation is needed into the mode of delivery and for a longer duration.

Benolea^®^ EFLA^®^943 (Olive leaf extract (*Olea europaea* L.), IFF Health, Londerzeel, Belgium) is a standardized dry olive leaf extract (DOLE) that has shown excellent pharmacological effects such as antioxidant, anti-obesity, and anti-hypertensive actions [127,128,129]. Goenka and Simon found potential as a pro-melanogenic candidate instead of an anti-melanogenic [127]. Previous studies have shown that olive leaf extracts have a strong antioxidative potential, and a study conducted found that OLE standardized to oleuropein levels had synergistic effects with UV filters and exerted improved photoprotective benefits, contributing to the in vitro sunscreen protection factor (SPF) of sunscreen formulations [130,131,132].

The use of phytochemicals in cancer therapy was also investigated; this study investigated the effect of DOLE on the progression of the highly malignant, immuno-, and chemoresistant form of skin cancer—melanoma [133]. The findings were the first to show anti-melanoma action in vitro and in vivo. Oleuropein, a significant component of DOLE (accounting for over 20% of the extract), has been shown to inhibit cancer cell proliferation, motility, and invasiveness. However, because cancer therapy and other anti-cancer medications are frequently used, combining DOLE with other cytostatic medications, such as doxorubicin or temozolomide, reduced their effectiveness, indicating a negative impact of these interactions [134,135]. With the management being troublesome with anticancer agents, preventative measures could be ideal as its main component, oleuropein, prevents chronic ultraviolet B radiation (UVB). It is postulated that the preventative effects of the olive leaf extract and oleuropein on chronic UVB-induced skin damage and carcinogenesis and tumor growth may be due to inhibition of the expression of VEGF, MMP-2, MMP-9, and MMP-13 through a reduction in COX-2 levels [136,137,138].

There is much more to discover and manipulate when treating cancers of different origins and immunological statuses.

## 13. Spirulina

Spirulina is a high-nutritional-value edible cyanobacterium, and interest in its biological activity and bioactive components, such as pigments, phenolics, peptides, and lipids, is growing [139]. Ikeda et al. undertook a comprehensive evaluation of spirulina potentials in dermatology; the overview focuses on the potential effects of spirulina or its separated constituents on the skin for either cosmetic or therapeutic goals. Spirulina and its components promote cutaneous fibroblast and keratinocyte proliferation, extracellular matrix, and collagen formation, as well as antioxidant and anti-inflammatory activity. The environment of the skin contributes to the reduction of cooperating for the emphasized anti-aging, photoprotection, and wound-healing benefits [140,141,142]. Spirulina is sold either as a supplement for daily use or in various products such as masks or creams. Because of its properties, its usage is beneficial for oily and acne-prone skin and also for dehydrated, mature skin.

In vitro, the assessment of spirulina extract mixed into skin cream for wound healing was the topic of a comparative investigation. The findings agreed to be helpful for both cosmeceutical and biological applications [143], as well as prospective uses to treat bacterial infections, herpes, and acne [139,144,145]. The evidence given indicates a lot of promise, but further study and testing are needed to fully understand its administration potential. Despite its many properties and rich composition, spirulina provides several advantages and displays.

Spirulina extract has been studied for its immunostimulatory effects on autoimmune skin pathologies such as dermatomyositis (DM), pemphigus/pemphigoid, and lupus erythematosus [146,147,148,149,150,151]. The background of spirulina’s wide health supplement increases both cytokine and chemokine participation to boost gene expression and Th1 lymphocyte stimulation [152,153,154,155]; because of spirulina’s capacity to stimulate an immune response, it can battle viral and cancer antigens/cells [156,157]. With studies indicating increased NK cell activity, there is little question that spirulina might contribute to more holistic approaches to cancer therapy and infection control [157]. A pilot trial used 200–400 mg/day every day for a week, and data revealed higher mRNA expression of immune cells, further boosting the supplement’s power [158,159]. More research and testing are required to demonstrate its value with a bigger population size. Some reports have also flagged up causing DM or exacerbated the onset of the pathology [147,148]. Many misunderstandings regarding herbal supplements exist since such bottles of extracts and minimal testing are subject to little oversight. Increased levels of TNF- may have a role in autoimmune disease flare-ups or aggravated DM because of the immunostimulatory effects. However, further study is needed to explain the causes of such events.

Acquired immune deficiency or HIV-infected patients have been using spirulina for 12 months, and based on its innate and adaptive enhancement, CD4+ cells were elevated and reduced viral load, and proven by in vitro studies increased T and B cell proliferation for up to 14 days after intake [156,158,159,160,161,162,163].

Dermatological surgery and skin excision are popular practices for removing proliferating lesions and leaving a wound/scar; a mouse investigation focused on employing spirulina protein at 2% and 4% concentrations [164]. The results indicated that after 3 days, there is an increase in the proliferation and migration of human fibroblasts, which are vital in the formation and remodeling of new tissues [165]. Myofibroblasts are important in skin wound healing, and levels of ɑ-SMA (myofibroblast biomarker) were higher in the epidermal growth factor (EGF) or spirulina-included groups than in the control group [166,167]. According to these findings, spirulina promoted wound healing by increasing the number of myofibroblasts.

Many researchers are attempting to add antioxidant phytochemicals into photoprotective cosmetic formulations and cosmetic items relevant to skin health and photoprotective aspects. For example, cosmetic appliances using spirulina show extremely excellent potential in SPF 30 products. After 84 days of therapy, antioxidant addition to the sunscreen formulation dramatically improved skin pigmentation, collagen breakdown on the dermis, and hence skin net elasticity compared to the sunscreen alone [168,169]. According to the safety assessment, the subjects did not show any adverse reaction, presenting good skin tolerance.

There is a lot of promise and evidence pointing towards the value of phytochemicals added to skin care products or oral supplements. However, in today’s world, many people have allergies and adverse reactions to skin products; thus, additional research into skin safety for application is needed.

## 14. Chlorella

Chlorella is a green unicellular alga that is commercially manufactured and marketed as a nutritional supplement all over the world. Chlorella products provide a variety of minerals and vitamins, including D and B12, that are not found in plant-based foods [170,171,172,173]. Chlorella provides more folate and iron than other plant-based meals [171]. Supplementing Chlorella to animals, including humans, has been shown to have a variety of pharmacological actions, including immunomodulatory, antioxidant, antidiabetic, antihypertensive, and antihyperlipidemic effects. It is sold in the form of supplements, often combined with spirulina extracts as well to facilitate the beneficial effects. Spirulina is also becoming a more popular ingredient for various cosmetics such as creams or masks.

Some of the advantages mentioned above include cholesterol reduction, atherosclerotic plaque prevention, and anti-tumor and antibacterial properties [146,174]. There have been no reports of Chlorella causing autoimmune diseases; nevertheless, various in vitro and in vivo tests show that it can activate the immune system [175,176]. Similarities with other phytochemicals, such as spirulina, can boost the Th1 cell response in both normal and immunocompromised hosts, as well as resistance to intracellular Listeria monocytogenes infection and NK cell activity [157,177].

According to a few studies, Chlorella increases collagen production in the skin, making it ideal for anti-aging and wound-healing [178,179,180]. Oral supplementation of Chlorella was found to reduce Dermatophagoides farinae (DFE)-induced atopic dermatitis (AD) in NC/Nga mice and capable of reducing drug-induced cancer by increasing the amounts of sulfhydryl (-SH) and glutathione S-transferase (GST) in the skin [181,182]. As the years pass, there is still a need to examine various skin types and sensitivities, and with the supplied information reviewed, prospective anti-inflammatory processes give rise to this promise.

Bax et al. conducted a review of environmental factors that initiate DM and incorporated herbal supplements and known medication often prescribed/treated [147,148]. There have been reports that such herbal supplements lead to DM, albeit the bottled components do not often clarify exactly what is in them. Little is known whether Chlorella plays a role in DM or other autoimmune diseases; perhaps more attention is needed to be able to identify and discriminate.

A case was raised in Japan to investigate whether herbal supplements produce erythema multiforme; the findings, according to evidence, the lymphocyte transformation test (LTT) is unreliable for in vitro supportive diagnosis of adverse events induced by natural food items containing more than one blended component due to constitutive false positives. As a result, further testing is required to differentiate herbal supplements given orally [183].

Further research studied against UVC-induced cytotoxicity. Here, human skin cells were treated with chlorella-derived peptide (CDP), vitamin C, or vitamin E after 15 J/cm^2^ of UVC irradiation [184]. UVC irradiation caused cytotoxicity in all groups except those treated with CDP, according to the findings. Caspase-3 activity was reduced in CDP-treated cells after 12 h. CDP protects against UVC-induced cytotoxicity by inhibiting caspase-3 activity and decreasing the levels of phosphorylated FADD and cleaved PARP-1. Not only UVC-induced skin cells but UVB-induced skin cells were also studied, and it was shown that CDP suppresses UVB-induced MMP-1 expression in skin fibroblasts by reducing the expression of AP-1 and CYR61, as well as MCP-1 production [185].

Skin cancer treatment has reappeared, thanks to a bioengineered Chlorella virus (Chlorella virus pyrimidine dimer glycosylase (Cv-pdg)) that improves penetration; formulated novel approach began with an enzyme that improves UV-damaged DNA repair, and this cytoplasmic enzyme can be used topically because it does not affect nuclear strictures [186,187]. Cv-pdg has been proposed to have broad effectiveness against a wide range of UV-induced mutations and, hence, in contrast to other putative chemopreventive medicines [186,187,188].

The majority of supplements are indicated to be used after a specific age; however, adolescent studies are frequently overlooked owing to being underage, accessibility, and parental agreement. Children are more prone to allergies and hypersensitivities than adults [189]. As more young people experiment in recent years, studies would benefit from the complete examination of the supplement sector.

## 15. Omega-3

Omega-3 fatty acids are a group of polyunsaturated fatty acids that are immensely common in nature and almost everyone’s diet. They are widely known for their positive influence on the human organism and therapeutic effects on such organs as skin. It has also long been known that Omega-3 acids play a huge role in inflammation or autoimmune processes. By recomposing cells’ membranes, modifying their metabolism, and many other mechanisms, the aforementioned acids contribute to healing a sizable group of diseases or preventing UV radiation [190,191,192,193]. Omega-3 acids are very common in topical cosmetics such as creams but also various supplements for internal use in the form of daily supplementation. According to some research, Omega-3 might be beneficial to facilitate the health and condition of the skin since it helps in the alleviation of inflammatory lesions on the skin and relieves the symptoms of dryness and irritation of the skin. Skin inflammatory diseases are the most popular pathologies, and Omega-3 PUFAs have confirmed the therapeutic effect. Psoriasis is an autoimmune chronic disease that affects humans’ skin and joints. Many factors can cause its activation or exacerbation [194]. It could expand all over the body and even lead to erythroderma (more than 90% of the skin area being reddened and desquamated) [195,196]. By replacing the amount of consumed saturated fatty acids with PUFAs, including Omega-3 acids, patients could, in all likelihood, reduce the progress of inflammation [197]. Another inflammatory disease that the Omega-3 acids family has an influence on is atopic dermatitis. Scientists may surely single out many prevention strategies for AD, but we cannot forget about the anti-inflammatory activity of these compounds. Omega-3 supplementation may be beneficial during therapy for atopic dermatitis, especially in children [198]. Research has shown that improvement of the skin barrier reduces the occurrence of AD [199,200]. What is more important is that the Omega-3 acids family might have a potential in cancer therapy. For example, their famous anti-inflammatory function leads to a decrease in breast cancer risk or even could help during the treatment of it. Furthermore, they reduce the complications associated with breast cancer presence, such as depression, pain, paraneoplastic syndromes, and eating disorders. Muscle weight loss as a result of chemotherapy might also be slowed down [201,202]. However, PUFAs are not only used in breast cancer therapy. For the same reasons as above, lung cancer supplementation with Omega-3 polyunsaturated acids can be supported. Research has shown promising factors of protective activity of them on the lungs [203,204]. Supplementation of marine Omega-3 acids can also reduce the risk of one of the most fatal diseases in the world, which is cardiovascular pathology. Using them in the case of prevention of hypertension or hypertriglyceridemia, which can lead to the appearance of cardiac diseases, has a therapeutic effect and may help reduce the risk of fatal consequences [205,206].

## 16. Astaxanthin

Astaxanthin (ASX) is a keto-carotenoid that is classed as a xanthophyll but is a carotenoid with no vitamin A action [207,208]. It is present in the majority of red-pigmented aquatic species [209]. Astaxanthin has been found to have antioxidant and anti-inflammatory properties. It may be found as a color additive in fish feed or certain animal feeds. Because of availability and demand in the pharmaceutical sector, ASX is mass-manufactured due to its murine biological source [210]. It is currently sold in various supplements as it was observed that astaxanthin might be helpful in the maintenance of the proper elasticity of the skin, reduction of wrinkles, and also facilitating the prevention of UVA and UVB.

ASX has been characterized as a dermatological therapeutic with effects on hyperpigmentation suppression, melanin production, photoaging inhibition, and wrinkle formation decrease in various clinical trials [208,211]. ASX possesses skin-protective antioxidant actions involving internal (chronological) and extrinsic (photo-) aging processes, such as the formation of reactive oxygen species (ROS) via oxidative metabolism and solar UV radiation exposure [208,212,213]. It has been demonstrated that ASX reduces UV-induced reactive nitrogen species generation, inflammatory cytokine expression, and apoptosis in keratinocytes. After UV irradiation, ASX significantly reduced the levels of inducible nitric oxide (iNOS) and cyclooxygenase (COX)-2, as well as the production of prostaglandin-E2 (PGE2) from keratinocytes [214]. The inhibition of iNOS synthesis by ASX has crucial implications for the development of anti-inflammatory medicines for cutaneous inflammatory illnesses, including psoriasis and atopic dermatitis. ASX reduced the gene expression of many proinflammatory indicators in an atopic dermatitis model, including interleukin-1 (IL-1), interleukin-6 (IL-6), and tumor necrosis factor-ɑ (TNF-ɑ) [215,216].

Other reports have emerged ASX to be immunostimulatory: in vitro studies on human lymphocytes have also shown that ASX increases immunoglobulin production in response to T cell-dependent stimuli, as well as increased natural NK cell cytotoxic activity, highlighting the importance of NK cell regulation in serving as an immunosurveillance system against tumors and virus-infected cells [217,218,219].

In animal trials, guinea pigs were given oral supplements of ASX of various Z-isomers [220]. On the seventh day, the animals’ dorsal foot pads were exposed to UV-induced irradiation. Elasticity, transepidermal water loss (TEWL), and pigmentation (melanin and erythema levels) were chosen as parameters to be examined. According to this study, dietary ASX accumulates in the skin and appears to protect against UV-induced skin damage, and the Z-isomers are more powerful oral sunscreen agents than the all-E-isomer.

Because UVB radiation is known to cause skin damage and promote skin cancer in the general population, studies are now testing natural substances on epidermal skin cells; Chung et al. pre-treated with astaxanthin for 24 h and exposed to UVB irradiation, the results were consistent with the ASX hypothesis [221].

In today’s world, modern cosmetic medicine is on the increase, with dermal fillers being one of the most well-known operations. ASX was used with polycaprolactone (PCL)-based microspheres in one research [222]. In vivo tests were conducted, and ATX was added to the microspheres for 16 weeks; favorable effects, such as tissue regeneration and collagen synthesis enhancement, were seen.

A daily regimen involves the application of cosmetics and sunscreens to guard against environmental changes, particularly the increase in UV radiation rate, which causes skin damage and premature aging. As previously stated, chemical UV filters are widely used in sunscreen formulations; nevertheless, they can also cause allergic responses. As a result, some pigments derived from microalgae, such as ASX, lutein, β-carotene, and other biocompounds [223,224], are now well described in the literature and biotechnologically manufactured as natural ingredients to be incorporated into skincare products with multifunctional benefits, including sunscreen [224,225].

Drug administration of active substances is one of the most difficult and advantageous ways to conventional pharmacology; numerous bases have been employed and analyzed, and the problem persists to this day. Self-nano-emulsifying drug delivery systems (SNEDDS) have been developed and tested to boost ASX skin penetration and target its antioxidant and anti-inflammatory capabilities to the epidermis and dermis [225]. When applied to ASX, this method for the improved skin penetration of a highly lipophilic molecule has the potential to develop topical formulations for UV protection, anti-aging, and inflammatory skin disorders [226,227].

When lesions are clinically concerning and the risks of scarring are high, major surgery, such as skin flaps, is the first-line therapeutic technique for skin defect repair. Gürsoy et al. looked at the influence of ASX on the survival of random pattern skin flaps. If taken in doses of more than 4 mg/kg, astaxanthin enhances flap viability and vascularization and can be utilized as an adjuvant drug [228]. This is promising, as other reporters have stated, but when it comes to dosing, whether the correct form is used should be further investigated for clarity and patient safety and efficacy.

The cytoprotective effects of ASX are unwarranted because of its health profile within the skin’s microenvironment. Researchers have come a long way to digress the necessary information about herbal supplements, and although they provide additional benefits dermatologically, there is still a larger scale of interest in the vehicle being transported into the several epidermal layers to dermal and subcutaneous layers. Drug efficacy and stability for both oral and topical use needs further investigation and needs to include adolescent and neonatal studies, which are highly lacking due to the immunological battle of skin disorders (Table 1).

## 17. Conclusions

Although certain supplements are not peer-reviewed and are merely suggestions, holistic and homeopathic approaches are important parts of a patient’s journey to prevent and eradicate sickness and disease for their overall well-being.

The active ingredients mentioned in this research may alleviate the symptoms of several skin inflammatory illnesses by simulating cytokine release, transcription processes, antioxidant properties, or microbiome modulation. There is little question that both macronutrients and micronutrients have a profoundly good influence on the skin barrier and provide several other advantages that should be researched further.

Clinically, the precise dose of a particular compound is frequently misinterpreted and potentially biased. Clinical dosage procedures will not be used by the regulatory authorities in charge of such supplements as they may confuse the general population. Because of their anti-tumorigenic properties, supplements such as vitamin E or Omega-3 acids may have a role during chemotherapy, as well as doses for maintaining cellular functionality and usability. A correlation between including these compounds in the diet and the aging of the skin can be observed because of their UV irradiation protection. Fernblock^®^ is an excellent candidate and example of specific compounds researched from extract-to-pill-to-patient, which provides enormous benefits in reducing risks for neoplasm and protecting from UV.

From a medical and clinical standpoint, one’s perception for dosing and administering specific supplements and compounds would be to educate consumers and patients from a dose-dependent nurture. To enable patients to consume supplements safely, physicians should advocate for safe use and accurate guidance. Because extracts of any of the aforementioned supplements would need to be properly titrated and may or may not require a vector or biding agent to enable sufficient absorption, there is no straightforward method for administering those supplements. Thus, further analysis and RCTs are needed to understand its essential vs. beneficial parity. What should further be taken into consideration is the fact that different supplements might cause adverse effects of various intensities. Therefore, they should be used with caution and considering other potential drugs that might affect their supplementation.

## Figures and Tables

**Table 1:** Benefits and side effects of the compounds described.

Name of Compound	Benefits	Side Effects
Vitamin A	Modulates the immune response Maintains homeostasis of epithelial tissues and mucosa through retinoic acid Anti-inflammatory mechanisms [229]	Skin irritation Erythema [230]
Vitamin C	Assists in antioxidant defense against UV-induced photodamage Induces collagen biosynthesis as a key promoter in formation Squalene vitamin C increases epidermal thickness Accelerates wound healing Helps in burn wound healing [231]	Skin pigmentation Hypopigmented hair Stinging Erythema Dryness [232]
Vitamin D	Anti-inflammatory and antiproliferative effects Inhibits the production of psoriasin and koebnerisin [233]	Weakness Fatigue Loss of skin turgor Dry mucous membrane [234]
Curcumin	Anti-inflammatory and antioxidant properties [235]	Nausea Diarrhea Allergic reactions Interactions with warfarin [236]
*Polypodium leucotomos*	Immunomodulatory properties Antioxidant defense Photoprotection [66]	Gastrointestinal symptoms Pruritus [237]
*Simmondsia chinesis*	Anti-inflammatory properties Boosts topical medication absorption Functions in the restoration of injured skin Speeds wound closure Stimulates collagen I production Enhanced antibacterial action Photoprotection [238]	Allergic reactions [239]
Biotin	Promotes the grow of nails and hair [240]	Possible interactions with, e.g., ciprofloxacin, azithromycin, and clarithromycin (biotin presents interactions with more than 70 different drugs) Teratogenicity hazards Interferes with thyroid and troponin tests [240]
Gamma Oryzanol	Antioxidant and skin anti-aging properties Prevents sola UV-related skin cancer [113]	Ameliorates insulin resistance and hyperlipidemia in rats [241]
Olive leaf extract	Antiviral, antibacterial, anti-inflammatory, and antioxidant activities Stimulates repigmentation in vitiligo [242]	Allergic reactions [124]
Spirulina	Antioxidant and anti-inflammatory activity Emphasizes anti-aging, photoprotection, and wound-healing [243]	Diarrhea Bloating Flatulence Edema Headache Muscle pain Skin redness Sweating [244]
Chlorella	Immunomodulatory, antioxidant, antidiabetic, antihypertensive, and antihyperlipidemic effects Increases collagen production in skin [170]	Nausea Diarrhea Abdominal cramping Flatulence Photosensitivity Interactions with warfarin [245]
Omega-3	Anti-inflammatory benefits Prevents from UV radiation Antihypertensive and antihyperlipidemic effects [246]	Diarrhea Nausea Abdominal pain Constipation Vomiting Fatigue [247]
Astaxanthin	Antioxidant and anti-inflammatory properties Cytoprotective effects [248]	Nausea Diarrhea [249]

## Abbreviations:

AD	atopic dermatitis
AMPs	antimicrobial proteins
ASX	astaxanthin
CDP	Chlorella-derived peptide
Cv-pdg	Chlorella virus pyrimidine dimer glycosylase
DM	dermatomyositis
EGF	epidermal growth factor
NEs	nanoethosomes
OLE	olive leaf extract
URI	upper respiratory illness
PL	*Polypodium leucotomos*
RELMα	Resistin-like molecule α
SFN	sulforaphane
SLE	systemic lupus erythematosus
SOD	superoxide dismutase
SPF	sunscreen protection factor
THC	tetrahydrocurcumin
UV	ultraviolet
